# Natural language processing techniques for studying language in pathological ageing: A scoping review

**DOI:** 10.1111/1460-6984.12870

**Published:** 2023-03-24

**Authors:** Gloria Gagliardi

**Affiliations:** ^1^ Department of Classical Philology and Italian Studies University of Bologna Bologna Italy

**Keywords:** dementia, digital linguistic biomarkers, natural language processing, pathological ageing

## Abstract

**Background:**

In the past few years there has been a growing interest in the employment of verbal productions as *digital biomarkers*, namely objective, quantifiable behavioural data that can be collected and measured by means of digital devices, allowing for a low‐cost pathology detection, classification and monitoring. Numerous research papers have been published on the automatic detection of subtle verbal alteration, starting from written texts, raw speech recordings and transcripts, and such linguistic analysis has been singled out as a cost‐effective method for diagnosing dementia and other medical conditions common among elderly patients (e.g., cognitive dysfunctions associated with metabolic disorders, dysarthria).

**Aims:**

To provide a critical appraisal and synthesis of evidence concerning the application of natural language processing (NLP) techniques for clinical purposes in the geriatric population. In particular, we discuss the state of the art on studying language in healthy and pathological ageing, focusing on the latest research efforts to build non‐intrusive language‐based tools for the early identification of cognitive frailty due to dementia. We also discuss some challenges and open problems raised by this approach.

**Methods & Procedures:**

We performed a scoping review to examine emerging evidence about this novel domain. Potentially relevant studies published up to November 2021 were identified from the databases of MEDLINE, Cochrane and Web of Science. We also browsed the proceedings of leading international conferences (e.g., ACL, COLING, Interspeech, LREC) from 2017 to 2021, and checked the reference lists of relevant studies and reviews.

**Main Contribution:**

The paper provides an introductory, but complete, overview of the application of NLP techniques for studying language disruption due to dementia. We also suggest that this technique can be fruitfully applied to other medical conditions (e.g., cognitive dysfunctions associated with dysarthria, cerebrovascular disease and mood disorders).

**Conclusions & Implications:**

Despite several critical points need to be addressed by the scientific community, a growing body of empirical evidence shows that NLP techniques can represent a promising tool for studying language changes in pathological aging, with a high potential to lead a significant shift in clinical practice.

**WHAT THIS PAPER ADDS:**

**What is already known on this subject**

- Speech and languages abilities change due to non‐pathological neurocognitive ageing and neurodegenerative processes. These subtle verbal modifications can be measured through NLP techniques and used as biomarkers for screening/diagnostic purposes in the geriatric population (i.e., digital linguistic biomarkers—DLBs).

**What this paper adds to existing knowledge**

- The review shows that DLBs can represent a promising clinical tool, with a high potential to spark a major shift to dementia assessment in the elderly. Some challenges and open problems are also discussed.

**What are the potential or actual clinical implications of this work?**

- This methodological review represents a starting point for clinicians approaching the DLB research field for studying language in healthy and pathological ageing. It summarizes the state of the art and future research directions of this novel approach.

## INTRODUCTION

Natural language processing (NLP)—that is, the interdisciplinary field that aims to get computers to perform tasks involving human language (Jurafsky & Martin, 2008: 1)—is gaining popularity among the medical community. The applications are varied, ranging from research to diagnostics and direct patient care (Locke et al., 2021).

Notably, during the past few years there has been a growing interest in the employment of verbal productions as *digital biomarkers*, namely objective, quantifiable behavioural data that can be collected and measured by means of digital devices, allowing for a low‐cost pathology detection, classification and monitoring (Gagliardi et al., 2021: 1). In particular, NLP techniques are increasingly used for clinical purposes in the geriatric population. Numerous research papers have been published on the automatic detection of subtle verbal alteration, starting from raw speech recordings and transcripts, and such linguistic analysis has been identified as a cost‐effective method for diagnosing cognitive deterioration due to dementia. Moreover, in the present day, this novel approach is spreading to the identification of other clinical or psychiatric conditions in the elderly population, such as cognitive dysfunctions associated with metabolic disorders (e.g., type 2 diabetes mellitus; cf. Imre et al., 2019), depression (De Souza et al., 2021) and dysarthria (e.g., due to Parkinson's disease; cf. Rahman et al., 2021). Actually, such a cross‐disciplinary approach presents a promising answer to the clinical challenge of cognitive assessment by combining objectivity (and reproducibility) in the evaluation process with unintrusiveness, speed and low cost.

## BACKGROUND

Speech and language are valuable sources of clinical information on cognitive status because they change depending on psychological distress (e.g., depressive symptoms; cf. Bernard et al., 2016; Tølbøll, 2019) and decline in parallel with neurodegeneration. A broad body of scientific literature suggests that linguistic deficits can be found in several neurodegenerative diseases (Boschi et al., 2017; Rahul & Ponniah, 2019), especially in dementia of the Alzheimer type, where language disruptions are common both at the early stages and in the full‐blown pathology (Szatloczki et al., 2015), in combination with episodic memory and visuospatial impairments. Additionally, changes in verbal competence are receiving special attention as early signs of cognitive decline, since they are frequently present even in the preclinical and prodromal phases of the disease (i.e., subjective memory complaints—SMCs; and mild cognitive impairment—MCI; cf. McCullough et al., 2019). Consequently, linguistic performances have been extensively examined in the neuropsychological domain, in both research and clinical contexts, concerning the diagnostic process (Taler & Phillips, 2008; Filiou et al., 2020).

However, the assessment of verbal skills through standardized psychometric instruments is time‐consuming and prone to human bias: Current pen‐and‐pencil tools often lack sufficient evidence of reliability to dementia patients (Krein et al., 2019). This is in line with the low validity of common screening tests, such as the Mini‐Mental State Examination (MMSE) (Folstein et al., 1975) in identifying subtle deficits in cognitive functioning (Mitchell, 2009; Creavin et al., 2016; Hwang et al., 2019; Arevalo‐Rodriguez et al., 2021).

An early diagnosis is a pivotal challenge to the promotion of the optimal management of cognitive frailty—irrespective of the reason—at both the individual and societal levels. Timely risk identification and a prompt, customized intervention might reduce the psychological burden of patients and caregivers, enabling the implementation of preventive measures and appropriate treatment. Besides, it represents a key strategy to contain the economic impact on social welfare and healthcare systems (Calzà et al., 2015).

In this background, automatic speech and language analysis through NLP methods has progressively acquired prominence. As stated by several scholars (Beltrami et al., 2018; de la Fuente Garcia et al., 2020; Petti et al., 2020), the usage of digital linguistic biomarkers (DLBs) has many advantages compared with classical paper‐and‐pencil tests. First, their computation is completely non‐intrusive, time‐effective and inexpensive. As such analysis does not require extensive infrastructure or medical equipment or laboratories, gathering information is easy and quick (König et al., 2018). These characteristics make DLBs particularly suitable as a life‐course assessment. Second, this methodology can be administered remotely since it is easy to integrate with the existing telemedicine solutions. As the COVID‐19 outbreak has shown, telehealth is of the utmost importance during extreme events, to allow optimal service delivery to fragile populations—such as elderly people—while reducing the risk of direct person‐to‐person contact (Bertini et al., 2022; König et al., 2021a). Finally, NLP techniques provide offline and online measures of cognitive activities underlying language production which would otherwise be impossible to be extracted and quantified by human operators (Gagliardi et al., 2021).

### Rationale of the study

In this scoping paper, peer‐reviewed studies on the application of NLP techniques to the analysis of language changes in ageing were identified, collated, compared and evaluated to provide a useful resource for researchers to inform best practice. In particular, our purpose is to summarize the state of the art on this topic—with a special focus on cognitive frailty detection—through the analysis of the following key issues:

- What classification task is performed? What is the specific goal thereof?
- What type of linguistic data are collected and analysed? How?
- What classification methods are usually applied? How are they evaluated?

For each of these questions, some challenges and open problems are discussed.

## METHODS

In the absence of formal guidelines on the design and reporting of scoping studies (such as the PRISMA statement for systematic reviews; cf. Moher et al., 2009) we followed the suggestions of Arksey and O'Malley (2005), Munn et al. (2018) and Mbuagbaw et al. (2020). Our goal was to map the shreds of evidence available in this emerging area, being as comprehensive as possible in identifying relevant primary studies but at the same time allowing the replicability of the search outcomes.

The investigations by Barragán Pulido et al. (2020), Clarke et al. (2021a), de la Fuente Garcia et al. (2020), Petti et al. (2020) and Martínez‐Nicolás et al. (2021) represent a valuable starting point for this work, since they provide a consistent critical review of the considerable amount of papers published on the topic.

To base our observations on a comprehensive knowledge of the topic, the literature search was conducted in the databases MEDLINE, Cochrane and Web of Science, using the following keywords:

(dementia OR Alzheimer OR mild cognitive impairment OR cognitive frailty) AND (language OR speech) AND (NLP OR detection OR identification).

All databases were accessed between 1 and 3 September 2021. An updated search was performed on 20 November 2021. Further, we screened the proceedings of the following major international conferences in the field from 2017 to 2021:

- Annual Meeting of the Association for Computational Linguistics (ACL) (55th–59th editions: Long papers, short papers, student research workshop, system demonstrations, workshops).
- European Chapter of the Association for Computational Linguistics (EACL) (15th and 16th editions: Long papers, short papers, student research workshop, software demonstrations).
- Asian Chapter of the Association for Computational Linguistics (AACL) (1st edition: Research papers, student research workshop, system demonstrations, workshops).
- North American Chapter of the Association for Computational Linguistics (NAACL) (2018, 2019 and 2021 editions: Research papers, student research workshop, demonstrations, workshops).
- International Conference on Recent Advances in NLP (RANLP) (2017, 2019 and 2021 editions).
- International Conference on Computational Linguistics (COLING) (27th and 28th editions: Research paper, system demonstrations, workshops).
- Interspeech (2017–21 editions).
- International Conference on Language Resources and Evaluation (LREC) (11th and 12th editions).
- Resources and Processing of Linguistic, Para‐Linguistic and Extra‐Linguistic Data from People with Various Forms of Cognitive/Psychiatric Impairments (RaPID workshop) (2nd and 3rd editions).

In addition, the reference lists of relevant studies were also checked, for further confirmation. We then applied some exclusion criteria: Only English‐language, peer‐reviewed articles were considered and pre‐print and unpublished works were not taken into account. Moreover, we discarded the papers lacking a clear description of the enrolled cohort. In the end, we based our observations on a sample of 179 papers.

## NLP TECHNIQUES FOR THE ANALYSIS OF LANGUAGE CHANGES DUE TO DEMENTIA: THE STATE OF THE ART

As mentioned in the Introduction, computational analysis of language changes due to ageing is performed through the estimation of DLBs. In this review, we will focus our attention on dementia assessment—and especially, among many proteins misfolding diseases, on Alzheimer's disease early identification—which has always been one of the most explored research areas in this field. However, this method can be seamlessly extended to a variety of clinical conditions, including but not limited to dysarthria, cerebrovascular disease and mood disorders (Gagliardi et al., 2021).

While the scientific literature documents a wide range of approaches to tackle the problem, it is possible to pinpoint at least some common steps:

- Collection (and eventual annotation) of verbal production written/uttered by patients and (matched) healthy controls.
- Automatic extraction of quantitative linguistic features and the optional preliminary testing of their discriminative powers.
- Application and validation of classification algorithms on these data.

Most of the time, studies adopt an observational retrospective case‐control setting (Mann, 2003).

Machine learning (ML)—the subfield of artificial intelligence (AI) that is concerned with the question of how to construct computer programs that automatically improve with experience (Mitchell, 1997: xv) in order to perform an automated detection of meaningful patterns in data (Shalev‐Shwartz & Ben‐David, 2014: xv)—is pivotal to this research program. In short, in DLB research, speakers’ data are usually annotated with labels for the target diagnosis (e.g., AD, Alzheimer's disease; MCI, mild cognitive impairment; SCI, subjective cognitive impairment; and HC/CON, healthy controls), established based on clinical evaluation (e.g., mainly neuropsychological testing, but including structural/functional brain imaging and fluid biomarkers). This information, known as the ‘ground truth’, is exploited to train the ML algorithm, so that it can ‘learn’ to discriminate the diagnostic classes in the training set. This procedure enables the system to predict the cognitive status of new, previously unseen speakers on a statistical basis, with some accuracy. Less frequently, an ‘unsupervised’ learning model is applied, in which case the algorithm is not provided with any pre‐assigned labels for the training and must seek to structure unannotated data, self‐discovering the occurrence of any hidden patterns (Jo, 2021).

We attempt to answer the research questions posed in the Rationale section by outlining the various phases involved in this procedure, from task definition to communicative/cognitive deficit identification and prediction, passing by cohort selection, data‐gathering and linguistic feature extraction.

### The DLB research field: Overall goals and task definition

The goals of the DLB research field are twofold: (1) to detect communicative impairments due to cognitive frailty in a screening/diagnostic perspective; and (2) to monitor the progression of linguistic symptoms throughout the disease trajectory.

Most of the works are devoted to the first aim: They try to distinguish the verbal productions of patients with a certain degree of cognitive impairment (i.e., subclinical, preclinical or full‐blown pathology) and stage of neurodegeneration (i.e., early, moderate, severe) from those of healthy controls. Generally, even when datasets include three or more different cohorts, most studies perform only pairwise comparisons: Dementia versus healthy ageing. However, this may be incorrect from a clinical application perspective, to create viable tools for real‐life environments. In fact, this dominant pairwise perspective does not grasp the complexity of cognitive frailty assessment in geriatric settings (Panza et al., 2015).

Just a few papers are devoted to fine‐grained dementia classification (e.g., targeting the underpinning protein misfolding diseases), all dealing with frontotemporal degeneration subtyping (e.g., Fraser et al., 2014; Garrard et al., 2014; Nevler et al., 2019; Themistocleous et al., 2018; 2021; Cho et al., 2020). Moreover, despite mood disorders—especially depressive symptoms and apathy—being very common in older adults, causing reversible deterioration of cognitive performances, researchers have dealt only occasionally with the assessment of behavioural noncognitive disturbances of patients (e.g., König et al., 2019, 2021b; Sumali et al., 2020; Villatoro‐Tello et al., 2021).

Limited progress has been achieved regarding the second goal too—that is, to follow the trajectory of the syndrome. To date, cross‐sectional paradigms are still the most popular across the community: Although some corpora do include longitudinal speech samples, researchers have not focused on predicting the conversion from subclinical or preclinical stage to full‐blown dementia, except in isolated cases (cf. Clark et al., 2016; Weiner & Schultz, 2016).

### Patient enrollment, data collection and DLB computation

A fundamental step of the DLB experimental approach is the recruitment of cohorts and linguistic data collection. Concerning this, papers should be explicit on inclusion/exclusion criteria for patient enrolment, following international guidelines (cf. Jack et al., 2011; McKhann et al., 2011; Albert et al., 2011; Sperling et. al., 2011; Dubois et al., 2014), to guarantee comparability with other studies. To the extent possible, the cohorts should be balanced—at least considering sex, age and education—since conclusions drawn from skewed corpora are subject to bias, especially in small datasets. Moreover, the statistical confirmation of cohort balance should be reported (as in Beltrami et al., 2018). Unfortunately, as observed by de la Fuente Garcia et al. (2020), not all research projects are in line with these basic design criteria.

DLBs can be detected both from written and oral texts, but the former approach is much less common (e.g., Toledo et al., 2014; Aramaki et al., 2016; Rentoumi et al., 2017). On the latter, different ‘speaking styles’ have been exploited: Read speech, repetition and (semi‐)spontaneous speech. Moreover, researchers tested either telephone (e.g., Tröger et al., 2018; Yu et al., 2018) or on‐site face‐to‐face recording set‐ups (e.g., in clinical settings or during natural conversations). Occasionally, avatars and virtual agents were used to trigger verbal productions (e.g., Tanaka et al., 2017; Ujiro et al., 2018; Mirheidari et al., 2019; O'Malley et al., 2021). A popular elicitation strategy is the recording of verbal productions during standardized neuropsychological assessment: In this domain, the most widely used tasks are ‘verbal fluency’ (phonemic or semantic) and picture description (e.g., the Cookie Theft picture from the Boston Diagnostic Aphasia Examination; cf. Goodglass et al., 1984). Conversely, (semi‐)spontaneous speech is usually triggered by interviewing the patients, engaging in conversation with them or asking them to recall something (e.g., a narrative plot, an event, a day or a dream). As the reader can observe, there is a high variability of study methods and settings in the scientific literature. Consequently, in our opinion, it is not possible to offer any advice on the more effective elicitation task at the moment. A deeper comparative analysis would be desirable, expanding the findings of Clarke et al. (2021b), Yamada et al. (2021) and Ivanova et al. (2022).

DLBs can be extracted directly from the audio files or the text/transcript. Transcription can be made manually or through automatic speech recognition (ASR) algorithms. The former strategy has reduced usability in the real‐life context, due to its time‐consuming and mistake‐prone nature. However, it is also true that open‐source ASR tools are not currently reliable for pathological speech automatic recognition.

Considering the DLB computation, there is a remarkable variability among the studies regarding extraction methods. Most researchers adopt their own algorithm for feature extraction (e.g., Calzà et al., 2021; Gagliardi & Tamburini, 2022), but the situation is set to change over the next year, thanks to the increasing number of open‐source tools and standardized feature sets (e.g., for acoustic indices, OpenSmile; Eyben et al., 2010). A striking number of features have been employed in the literature as proxy measures of cognitive disorder due to dementia. These can be classified into three main groups:

1. *Speech‐based features* comprise DLBs directly extracted from audio samples. They convey both linguistic and paralinguistic information (i.e., vocal cues expressing emotions, irony, etc.). They can be further split into:
  - *Acoustical* (e.g., López‐de‐Ipiña et al., 2015; 2018; Haider et al., 2020):
    - *Prosodic features*, concerning temporal properties of the speech (e.g., pause rate, phonation rate, speech rate, articulation rate) and fundamental frequency (F_0_).
    - *Loudness and energy*.
    - *Spectral features*, such as formant trajectories (i.e., F1, F2 and F3), mel frequency cepstral coefficient (MFCC) and spectral centroid.
    - *Vocal quality*, such as jitter, shimmer and harmonic‐to‐noise ratio (HNR).
    - *ASR‐related features*, such as disfluencies, repetitions, filled pauses and fractal dimension.
  - *Rhythmic*, that is, variability of the syllabic intervals (cf. Martínez‐Sánchez et al., 2016; Meilán et al., 2020; Calzà et al., 2021).
2. *Text‐based features* consist of DLBs computed on written texts or transcripts. They gauge a wide range of linguistic dimensions, at multiple levels:
  - *Lexicals* are DLBs which probe vocabulary richness (e.g., type‐token ratio, Brunét's index, Honoré’s statistics), the ‘density’ of verbal productions (e.g., content density, idea density), the rate of part of speech (e.g., nouns, verbs, adjectives, pronouns) or the incidence of specific lexical–semantic categories (cf. LIWC (Linguistic Inquiry and Word Count) ; Tausczik & Pennebaker, 2010).
  - *Syntactical DLBs* measure the complexity of sentence structure on constituency‐ or dependency‐based parse trees (cf. Roark et al., 2011; Lundholm Fors et al., 2018).
  - *Semantic DLBs* explore the meaning of the texts (e.g., through matrix decomposition methods such as Latent semantic analysis (LSA) and Principal Component Analysis (PCA) , embeddings, topic modelling and sentiment analysis).
  - *Pragmatic DLBs* quantify the usage of deictics and the coherence of the text.
3. *Extra‐linguistic/multimodal features* such as gaze (e.g., Fraser et al., 2019a), smile (e.g., Tanaka et al., 2017) and gait (e.g., Shinkawa et al., 2019) are collected through wearable devices or sensors.

See Voleti et al. (2010: 284), de la Fuente Garcia et al. (2020: 1552) and Petti et al. (2020: 1791) for a detailed overview of the indices and their presumed discrimination power in dementia research. However, it is critical to highlight that the actual occurrence of specific linguistic cues in relation to different age‐related cognitive profiles is under debate (Gagliardi & Tamburini, 2022). Language alterations brought on by dementia have been mostly reported in the lexical, syntactic and pragmatic domains. In a clinical perspective, they can be easily explained by the typical localization of brain atrophies (e.g., medial temporal lobe and hippocampus for AD) and linked to other cognitive alterations (e.g., episodic and semantic memory, executive functions). As a result, the computational analysis of these verbal competencies can be considered a reliable, well‐established source of DLBs. However, it should be emphasized that algorithms usually display even higher performance on acoustical data (i.e., segmental and prosodic features of the speech, probably linked to anatomically driven phenomena). Unfortunately, the nature and the clinical relevance of these voice abnormalities remain largely unexplored. A larger body of evidence is needed to shed light on this point.

To conclude this section, we would like to emphasize that large linguistic corpora are essential for this research domain. However, their collection, filing, sharing and dissemination raises several ethical and legal issues that affect their full accessibility to the scientific community (e.g., pathological speech recordings and DLBs pertain to ‘special category of personal data’ according to the European Union's (EU) General Data Protection Regulation (GDPR), which imposes strict privacy rules). Several initiatives, such as the DELAD project (Lee et al., 2021), are currently in progress to overcome these problems. Nonetheless, currently, data scarcity represents one of the main limitations for the generalizability of the findings and translation of this technique into clinical practice.

### Classification through ML methods: Adopted algorithms and their evaluation

A large variety of algorithms have been applied to the task, ranging from ‘conventional’ supervised classifiers to deep learning methods (see Aggarwal, 2019; and Jo, 2021, for a comprehensive picture of the field). The choice mostly depends on the dataset size.

The conventional supervised algorithm includes naïve Bayes classifiers, *k*‐nearest neighbour, logistic regression, support vector machine (SVM), random forest (RF) or algorithms that produce interpretable outputs such as decision trees, applied singly or in combination. Since researchers usually deal with small datasets, a smaller number of studies have used artificial neural networks and deep learning methods, such as convolutional neural networks (CNNs), long short‐term memory networks (LSTMs), recurrent neural networks (RNNs), multilayer perceptrons (MLPs) and autoencoders. See de la Fuente Garcia et al. (2020: 30–41, supplementary material) for a detailed survey. Very recently, some research groups adopted a transfer learning‐based approach and language models (Yang et al., 2020), such as bidirectional encoder representations from transformers (BERT) (Devlin et al., 2019), with encouraging results (Haulcy & Glass, 2020; Balagopalan et al., 2021; Guo et al., 2021; Liu et al., 2021; Roshanzamir et al., 2021; Saltz et al., 2021). The more promising aspect of this novel methodology is the possibility of eliminating the time‐consuming step of feature engineering and mitigating the need for a big dataset. However, along with other deep learning models, it raises an issue known as the ‘black box’ problem: Unlike feature‐based approaches, these systems are ‘opaque’, that is, it is difficult to ‘look inside’ to explain their behaviour (Zednik, 2021: 1). In other words, it is not clear what information (i.e., set of features) in the input dataset determines the results. This point is very critical in a clinical setting because physicians should always be able to explain how a diagnosis was posed (Dashwood et al., 2021).

Building reliable ML classifiers is a challenging task. A decisive point is avoiding ‘overfitting’, that is, the model's inability to generalize to unseen data. In short, the algorithm performs well on the training set, but its results drop significantly when tested on unfamiliar data, because of the limited size of the dataset, its low quality or the task difficulty.

In this sense, the main criticalities arise from the validation of ML systems and their evaluation (including reporting strategies). On the first matter, following de la Fuente Garcia et al. (2020), the most established practice for testing the classifier is ‘cross‐validation’ (CV) which consists of randomly splitting the dataset into equal ‘folds’ (i.e., segments), using different portions to train and test the model on subsequent iteration. According to the authors, CV is probably the best choice in this context of data scarcity, since conventional hold‐out strategies would involve running the test on only a few observations. However, most studies have used the CV as an evaluation technique, but with a preliminary feature filtering (e.g., statistical comparison among the group by inferential tests or application of feature selection/dimension reduction methods, such as information gain, principal component analysis and minimum redundancy maximum relevance). In our opinion (Calzà et al., 2021), the aforementioned operation should be avoided because it artificially inflates the final performance feeding the algorithm with features picked beforehand, from the whole dataset (without dividing training and test sets).

Regarding the evaluation procedures, despite the growing volume of research on DLBs, a good practice for reporting the performances of ML systems has not yet been established across the NLP community. The choice of the evaluation metric is not a clear‐cut issue for this task (Gosztolya et al., 2019; Calzà et al., 2021). Results are sometimes reported by providing the receiver operating characteristics (ROC) curve, which plots sensitivity versus specificity across a range of values for the power to predict a dichotomous outcome or the area under the curve (AUC) and the equal error rate (EER). These two metrics descend from the ROC: They correspond, respectively, to the area subtended by the curve, and the point where the false‐positive rate and the false‐negative rate are equal, that is, the intersection of the ROC curve with the straight line of 45°. Several classical information retrieval metrics are also widely used: ‘accuracy’ (i.e., the number of correctly predicted samples over the total number of samples), ‘precision’ (i.e., the fraction of relevant samples among the retrieved samples) and ‘recall’ (i.e., the fraction of the total amount of relevant samples retrieved). These last two scores are usually combined in the ‘F‐measure’ (or ‘F1‐score’), which corresponds to their harmonic mean. However, many studies have reported accuracy alone and this can be misleading, especially in the case of a class imbalance (Calzà et al., 2021).

The heterogeneity of the reporting strategy makes the results hardly comparable. This situation, combined with the extremely rapid development of the field, discourages us from proposing a comparison among systems’ performances (which exceed 90% accuracy in AD detection but are significantly lower with MCI, around 75–80%). In this respect, Petti et al. (2020), Martínez‐Nicolás et al. (2021) and especially de la Fuente Garcia et al. (2020) provide an extensive and updated overview.

## DISCUSSION AND CONCLUSIONS

In this work, we reviewed the current approaches to language description and evaluation through NLP techniques in the elderly. In particular, we discussed the state of the art on studying verbal productions in healthy and pathological ageing and the latest research efforts to build non‐intrusive language‐based tools for the early identification of cognitive frailty due to dementia.

Summing up, a huge body of empirical evidence shows that automatic analysis of speech and language can represent a promising tool, with a high potential to spark a major shift to clinical practice. However, several concerns need to be addressed.

Unfortunately, the quality and quantity of information are currently insufficient to offer recommendations on the selection of tasks, features and algorithms. To date, as already observed by de la Fuente Garcia et al. (2020), it is unfair to compare on an equal footing the accuracy of ML methods, since their conclusions are drawn from data triggered by various elicitation techniques under different recording conditions, and the linguistic parameters are generated in a non‐standardized way (i.e., with extraction procedures poorly and inconsistently described, avoiding replication studies). On the first point—that is, the lack of comparability due to datasets—it would be advisable to systematically compare the different approaches against balanced benchmarks adopting common metrics (Luz et al., 2021a), such as the shared task provided by ADReSS Challenges (Luz et al., 2020; 2021b) at InterSpeech conferences. However, this aspect is quite challenging for languages other than English, and especially for underrepresented languages. This is not a minor issue, since typological peculiarities (at the acoustical, morphological, syntactic and lexical levels) may strongly affect the comparability and transferability of results. To date, only a few papers adopted a multilingual approach, to identify DLBs that can generalize beyond a single corpus/language model (e.g., Fraser et al., 2019b; Gosztolya et al., 2021; Lindsay et al., 2021).

In our opinion, at the theoretical level, some general issues should also be considered. As previously stated, most of the works are devoted to the discrimination between subjects with a clear diagnosis of dementia and healthy controls, in a binary classification setting. However, this task is barely helpful from a clinical perspective (Calzà et al., 2021; Gagliardi & Tamburini, 2021): According to the World Health Organization (WHO) (2017), national programs should promote early diagnosis to foster healthy lifestyles and enable adequate treatment. Therefore, we would like to recommend focusing future research efforts on pre‐clinical state detection, namely MCI or SCI.

Moreover, differential diagnosis, psychiatric comorbidities and longitudinal analyses should be a priority in future investigations. As a possible point of interest, a more granular description of MCI subtypes in the cohorts (i.e., amnestic/non‐amnestic, simple‐/multiple‐domain) (Winblad et al., 2004) should be provided, since each of them may have different aetiologies (e.g., degenerative, vascular, psychiatric, medication side effects) and progressions (Petersen, 2004).

Nevertheless, *repeatability*—namely, the average variation over a short period in which disease‐related decline is unlikely to be evident or manifested through the features (Stegmann et al., 2020:110)—should be considered seriously. It is crucial to distinguish normal variation in verbal productions (i.e., linked to stress, exhaustion, mood and motivation to perform the task) from disease‐related linguistic changes, to build a reliable tool.

As evidenced by Pessin et al. (2017) and Rojas et al. (2020), the acoustical properties of the voice and the rhythmical aspects of the speech are not only altered by the disease progression, but also change over a lifetime, because of some physiological events that occur in senescence (i.e., changes in the speech mechanism affecting the respiratory, phonatory and supralaryngeal systems, such as the weakening of respiratory muscles, ossification of laryngeal cartilages and atrophy of facial, mastication and pharyngeal muscles). These voice disorders, known as ‘presbiphonia’, manifest perceptually to the point that listeners can judge a speaker's age fairly accurately from speech alone (Pettorino & Giannini, 2011). Analogous considerations can be made on the lexical–semantic and formal aspects of language (Peelle, 2019; Poulisse et al., 2019; Wulff et al., 2019). To address these hurdles and adequately capture the disease trajectory considering age effects and demographic factors, a more accurate stratification of cohorts is required. Potentially relevant features may include socio‐economic status (SES) (APA, 2007) and cognitive reserve (Pettigrew & Soldan, 2019), namely the adaptability (i.e., efficiency, capacity, flexibility, etc.) of cognitive processes that helps to explain differential susceptibility of cognitive abilities or day‐to‐day function to brain ageing, pathology or insult (Stern et al., 2020: 1306). Further, dataset collection and system training should deal with diatopic variation, considering both cross‐ and intra‐linguistic differences (i.e., dialects and regional varieties spoken by the patients): Actually, this aspect represents one of the crucial problems for implementing a real large‐scale tool (Barragán Pulido et al., 2020). We feel that these pieces of information will provide new insights into understanding the origin of the communicative impairment, its nature (e.g., phonatory, articulatory or cognitive), and will also support the discrimination of different aetiologies, boosting the sensitivity and specificity of the methodology.

The verbal alterations detected by ML classifiers are loosely ascribed to cognitive abnormalities, despite the lack of clarity about its origins. The changes are usually explained through a decrease in motor control, deterioration of mnemonic systems and deficits of executive functions, but to date, robust and trustworthy correlations are yet to be presented. It would hence be interesting to investigate the neurobiological basis of linguistic biomarkers by linking deviant linguistic features with local brain atrophies or neural activity, combining (functional) neuroimaging studies and NLP methods.

Last but not the least, while most of the papers on the topic stress the potential of DLBs to outperform conventional screening approaches, only a few studies effectively implement the methodology in clinical research and medical practice (e.g., Martinez de Lizarduy et al., 2017; Martínez‐Sánchez et al., 2018; König et al., 2021a; Rentoumi et al., 2021; Liang et al., 2022). To quote de la Fuente Garcia et al. (2020: 1548), despite progress in research, the small, inconsistent, single‐laboratory and non‐standardized nature of most studies has yielded results that are not robust enough to be aggregated and thereafter implemented toward those goals. This has resulted in gaps between research contexts, clinical potential and actual clinical applications of this new technology. DLBs, similar to other standard medical devices, will require specific developmental stages to be considered reliable and safe for use. A first, crucial step in this direction may be to move from the adoption of this approach in case‐control designs to prospective investigations.

## CONFLICTS OF INTEREST STATEMENT

The author does not have any known competing financial interests that could have influenced the results reported in this paper.

## Data Availability

The full bibliography that supports the arguments presented in this study is available from the author, upon request.
